# Substrate-Modulated Electric and Magnetic Resonances of Lithium Niobite Nanoparticles Illuminated by White Light

**DOI:** 10.3390/nano12122010

**Published:** 2022-06-10

**Authors:** Hui Li, Yigeng Peng, Ruifeng Lu

**Affiliations:** Institute of Ultrafast Optical Physics, Department of Applied Physics & MIIT Key Laboratory of Semiconductor Microstructure and Quantum Sensing, Nanjing University of Science and Technology, Nanjing 210094, China; huili@njust.edu.cn

**Keywords:** nanoparticle, particle-film interaction, scattering spectra

## Abstract

The manipulation of light at the nanoscale is important for nanophotonic research. Lithium niobite (LiNbO_3_), as an ideal building block for metamaterials, has attracted great interest for its unique properties in the field of nonlinear optics. In this paper, we numerically studied the effect of different substrates on the optical resonances of a LiNbO_3_ nanoparticle. The results show that the electric and magnetic resonances of such a system can be effectively adjusted by changing the substrate. Compared to the impact of dielectric substrate, the interaction between the LiNbO_3_ nanoparticle and the Au film shows a fascinating phenomenon that a sharp resonance peak appears. The multipole decomposition of the scattering spectrum shows that the size, shape of the LiNbO_3_ nanoparticle, and the thickness of the SiO_2_ film between the particle and the Au film have a significant impact on the electromagnetic resonance of the LiNbO_3_ nanoparticle. This work provides a new insight into LiNbO_3_ nanoparticles, which may have potential use in the design of dielectric nanomaterials and devices.

## 1. Introduction

The study of light scattering of subwavelength particles can be traced back to 100 years ago when the Mie theory was provided and widely used to describe the optical resonance of spherical particles [1]. The electric and magnetic resonances of subwavelength particles have attracted tremendous interest as viable potential alternatives to nanophotonic devices.

Metal nanoparticles, for example, can support surface plasmons, which can be used to concentrate light into subwavelength volumes and produce higher optical intensity [2,3,4,5,6], thus paving the way to break the diffraction limit down to the nanometer scale. These extraordinary properties can trigger numerous fascinating optical phenomena such as superlensing effects [7,8] and surface-enhanced Raman scattering [9,10]. However, the majority of these undergo Ohmic loss and lack of magnetic resonance, which inevitably limit their application. By comparison with metal nanoparticles, dielectric nanoparticles possess lower dissipative loss for the small imaginary part of the refractive index. It was not until 2011 that dielectric nanoparticles with significant electromagnetic response in the visible and infrared wavebands were theoretically studied [11]. Dielectric nanoparticles with a high refractive index above 3.0, such as silicon (Si) [12,13,14], germanium (Ge) [15,16], and gallium arsenide (GaAs) [17], exhibit well-separated electric and magnetic dipole (ED and MD) resonances in the scattering spectra. It has been demonstrated that ED and MD resonances can be effectively controlled by adjusting the particle geometry and dielectric environment [18,19]. The circular displacement currents excited by the incident light lead to the oscillating dipole, termed as MD resonance. MD resonance occurs when the wavelength of the incident light (λ) and the diameter (D) of the particle have the relationship of λ = nD, where n is the refractive index of the particles. These nanoparticles can be utilized as building blocks for nanoantenna in the visible range. In contrast to the high refractive index nanoparticles, the nanoparticles with a refractive index around 1.7–3 also support ED and MD resonances [20,21,22,23,24,25,26]. In this case, the spectra of the ED and MD resonances tend to largely overlap with each other, which enables forward scattering to occur at the peak of total scattering spectra.

Unlike ideal studies of the scattering spectra in a free space, nanoparticles always need to be supported by various substrates that, in general, modify their ED and MD resonances. Thus, another degree of freedom for manipulating their optical properties is provided. So far, systematic studies of the interaction between nanoparticles and substrates have been carried out [2,3,4,5,22,27,28,29,30]. It has been theoretically and experimentally demonstrated that the dielectric substrates have a slight influence on the ED and MD resonances [30]. In contrast, the localized plasmon resonance of metallic nanoparticles and substrate is sensitive to the shape and size of the nanoparticle and the surrounding environment [4,27]. An interesting case is high refractive index dielectric nanoparticles placed on metallic substrate. The strong coupling effects lead to a significant enhancement in the electromagnetic field [29,30,31].

LiNbO_3_, as an important nonlinear material, has high second-order susceptibility coefficients, which can be widely used in electro-optical modulator [32,33,34,35] and phase modulator [36,37,38,39] devices, and in acoustic filters [40,41]. However, little attentions has been paid to the scattering properties of the LiNbO_3_ nanoparticle.

In this study, we calculate multipole decomposition of the scattering spectra of a LiNbO_3_ nanoparticle placed on different substrates by using a finite-different time-domain (FDTD) technique. Compared to a LiNbO_3_ nanoparticle supported on dielectric substrates, a sharp resonance mode appears, which is caused by the interaction of the LiNbO_3_ particle and the Au film. Furthermore, the evolution of the scattering spectra of LiNbO_3_ nanoparticles with different diameters is studied. It is demonstrated that the thickness of SiO_2_ film between the LiNbO_3_ nanoparticle and the Au film has an impact on the scattering spectra. Furthermore, the results show that the shape also has a great influence on the scattering mode.

## 2. Theoretical Analysis

The scattering spectra of the LiNbO_3_ nanoparticles in this work are calculated using the FDTD technique. Firstly, we calculate the background field without the presence of the LiNbO_3_ nanoparticle. The total scattering spectra of the LiNbO_3_ nanoparticle is then derived. Finally, the scattering spectra is obtained from the difference between the total scattering spectra and the background field.

To analyze the electric and magnetic resonances of the LiNbO_3_ nanoparticles placed on different substrates, we employ the multipole decomposition of polarization $P=\varepsilon_{0}\left(\varepsilon_{P}-\varepsilon_{d}\right)E$, in the Cartesian coordinate, where $\varepsilon_{0}$, $\varepsilon_{p}$, and $\varepsilon_{d}$ represent the vacuum dielectric constant, relative dielectric permittivity of the LiNbO_3_ nanoparticle, and relative dielectric permittivity of the surrounding medium, respectively. E is the total electric field inside the LiNbO_3_ nanoparticle. The multipole moment can be obtained by integration of the induced polarization currents over the volume of the LiNbO_3_ nanoparticle. As a result, the ED moment, electric quadrupole (EQ) moment, MD moment, and magnetic quadrupole (MQ) moment can be expressed as:

(1) $$\mathrm{ED}=\int P\left(r\right)dr$$

(2) $$\mathrm{EQ}=3\int \left[rP\left(r\right)+P\left(r\right)r-\frac{2}{3}\left[r\cdot P\left(r\right)\hat{U}\right]\right]dr$$

(3) $$\mathrm{MD}=-\frac{i\omega }{2}\int \left[r\times P\left(r\right)\right]dr$$

(4) $$\mathrm{MQ}=\frac{\omega }{3i}\int \left\{\left[r\times P\left(r\right)\right]r-r\left[r\times P\left(r\right)\right]\right\}dr$$

where ω is the angular frequency of the incident light, *r* describes the radius vector of a volume element inside the LiNbO_3_ nanoparticles, and $\hat{U}$ is a 3 × 3 unit tensor. The scattering cross sections of the ED, EQ, MD, and MQ can be expressed as:

(5) $$\sigma_{ED}=\frac{k_{0}^{4}}{12\pi \varepsilon_{0}^{2}v_{d}\mu_{0}}{\left|\mathrm{ED}\right|}^{2}$$

(6) $$\sigma_{EQ}=\frac{k_{0}^{6}\varepsilon_{d}}{1440\pi \varepsilon_{0}^{2}v_{d}\mu_{0}}\sum_{\alpha \beta }{\left|{\mathrm{EQ}}_{\alpha \beta }\right|}^{2}$$

(7) $$\sigma_{MD}=\frac{k_{0}^{4}\varepsilon_{d}}{12\pi \varepsilon_{0}v_{d}}{\left|\mathrm{MD}\right|}^{2}$$

(8) $$\sigma_{MQ}=\frac{k_{0}^{6}\varepsilon_{d}^{2}}{160\pi \varepsilon_{0}v_{d}}\sum_{\alpha \beta }{\left|{\mathrm{MQ}}_{\alpha \beta }\right|}^{2}$$

Here, *k*_0_, *v_d_*, and $\mu_{0}$ denote the wave number in vacuum, the speed of light in the surrounding medium, and the vacuum permeability, respectively. The subscript characters α and β represent x, y, z. The total scattering intensity (P*_sc_*) can be obtained by the superposition of the scattering cross sections of multipole moments. Note that the poles that are of a higher order than the quadrupole make less contributions to the scattering intensity [20,42]; here we include the contributions from ED, EQ, MD, and MQ, which can be expressed as:

(9) $$P_{sc}\approx \sigma_{ED}+\sigma_{MD}+\sigma_{EQ}+\sigma_{MQ}$$

The dielectric constants of LiNbO_3_, SiO_2_, and Au are respectively taken from Zelmon [43], Gao [44], and Rakić [45], while we assume that the refractive index of the glass substrate is equal to 1.5.

## 3. Results

**Figure 1:**
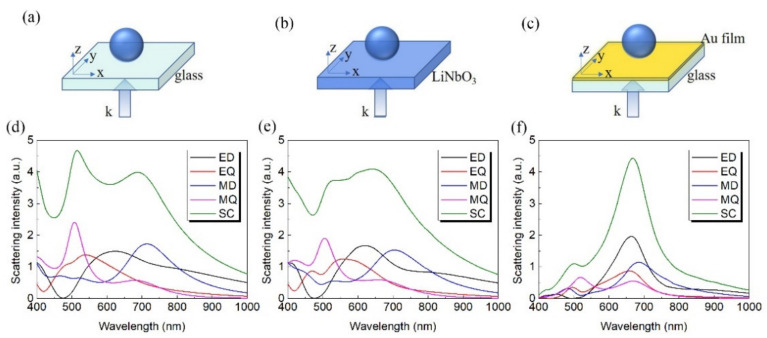
Schematic of LiNbO_3_ particle with diameter D = 300 nm placed on a glass substrate (**a**), a LiNbO_3_ substrate (**b**) and an Au film/glass substrate (**c**). The corresponding multipole decomposition of the scattering spectrum are displayed in the second row (**d**–**f**). SC represents the total scattering intensity.

**Figure 2:**
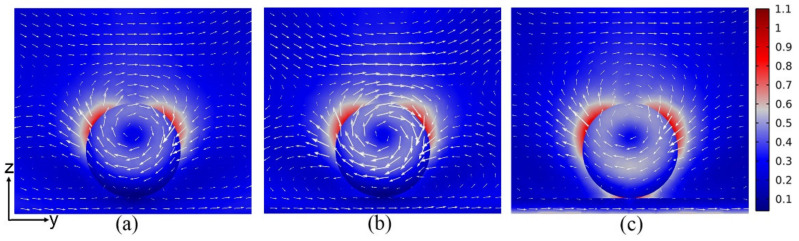
Electric field distributions in the yz plane at different peaks for 620 nm on glass substrate (**a**), LiNbO_3_ substrate (**b**), and for 665 nm on Au film/glass substrate (**c**). The white arrows depict the electric field vector.

**Figure 3:**
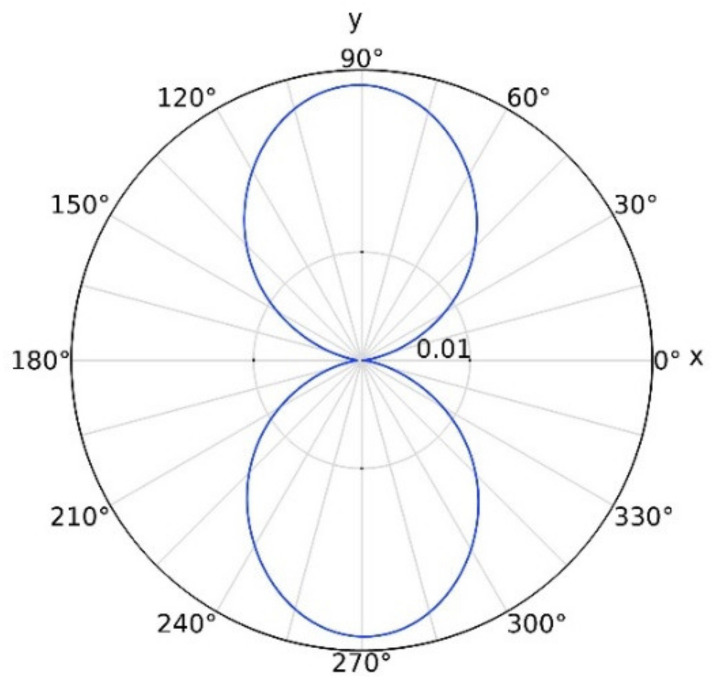
Radiation pattern in the xy plane for LiNbO_3_ nanoparticles (D = 300 nm) placed on an Au film/glass substrate.

In Figure 1, we present the total scattering spectra, as well as the contributions from multipole resonances calculated for a LiNbO_3_ nanoparticle located on top of the glass substrate, LiNbO_3_ substrate, and 50 nm Au film/glass substrate. A uniform environment with a refractive index of 1.0 is employed. The first row of Figure 1 shows the schematic of LiNbO_3_ nanoparticles with diameter D = 300 nm placed on different substrates. With the light incidents along the z axis and the polarization direction along the y axis, the corresponding multipole decomposition of the scattering spectra is calculated by using the FDTD simulation with respect to the center of the LiNbO_3_ nanoparticle, as shown in the second row of Figure 1. These results clearly demonstrate that ED resonance of LiNbO_3_ nanoparticles largely overlaps with that of MD resonance. Different from the conventional Si and GaAs nanoparticles, the EQ and MQ resonances of LiNbO_3_ nanoparticle also make a prominent contribution to scattering spectra, except for the ED and MD resonances. Note that with the increasing of the refractive index of the substrate, the ED resonance is enhanced while MD resonance is weakened, as shown in Figure 1d,e. More importantly, a significantly reduced linewidth is found for the LiNbO_3_ nanoparticles positioned on Au film (Figure 1f) compared to that on dielectric substrates (Figure 1d,e). The corresponding electric field distributions on the yz plane are depicted in Figure 2. It is clear to see that the peak of the scattering spectra is obvious for the Au film substrate (Figure 1f). On the contrary, the peaks of the other cases are not so pronounced (Figure 1d,e); here we show the electric field distribution at the peak of the ED. As shown in Figure 2c, a hotspot is generated between the nanoparticle and the Au film. Generally, for the LiNbO_3_ nanoparticle-metal film system, the hotspot is attributed to the interaction of the original ED and MD resonances of the nanoparticle and their mirror images aroused by Au film. According to the mirror image theory [31,33], the original ED mode induced by incident light leads to an antiparallel mirror ED mode inside the Au film, while the original MD mode results in a mirror MD mode inside the Au film. Due to the coherent interaction of the ED and MD resonances and their mirror images, the ultimate ED resonance of the LiNbO_3_ particle/Au film system is enhanced while the MD resonance is decreased. The far-field radiation pattern in the xy plane is depicted in Figure 3. It is remarkable that the strong power distribution is along the y direction, which indicates the existence of the dipole oscillating along the y direction.

**Figure 4:**
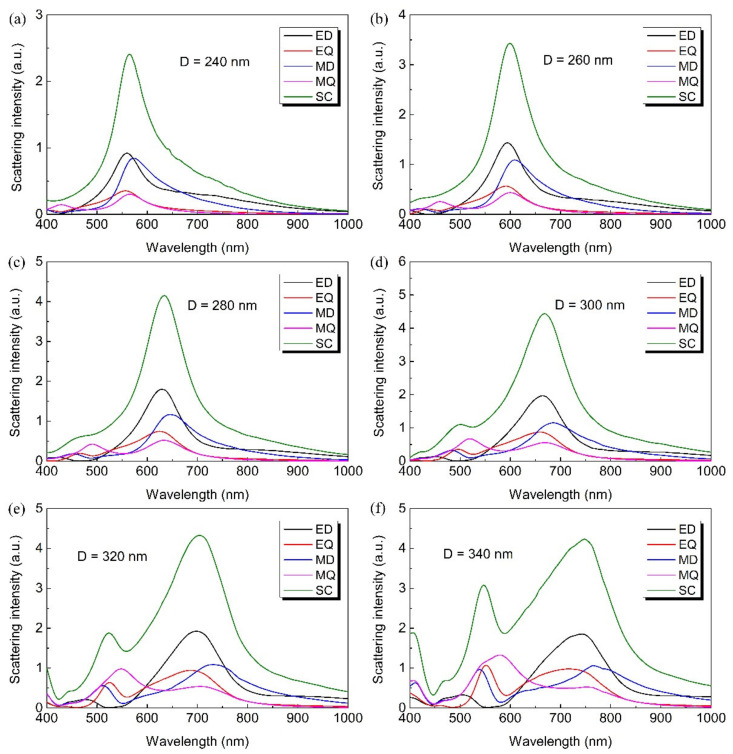
The calculated multipole decomposition of the scattering spectra of LiNbO_3_ particle with diameters (**a**) D = 240 nm, (**b**) D = 260 nm, (**c**) D = 280 nm, (**d**) D = 300 nm, (**e**) D = 320 nm and (**f**) D = 340 nm on Au film/glass substrate.

To corroborate this mechanism for the LiNbO_3_ nanoparticle on the Au film/glass system, we calculated the total scattering spectra, as well as the multipole decompositions of a LiNbO_3_ nanoparticle with different diameters. Figure 4a–f show the calculated spectra for a LiNbO_3_ nanoparticle with diameter D = 240 nm, 260 nm, 280 nm, 300 nm, 320 nm, and 340 nm, respectively. We can clearly see that the scattering spectra redshifts slightly and becomes wider when the diameter of the nanoparticle increases. At the same time, the ED resonance is strengthened while the MD resonance is weakened. An impressive phenomenon is that the quadrupolar resonances is enhanced, which may be caused by the symmetry breaking aroused by the presence of the Au film.

**Figure 5:**
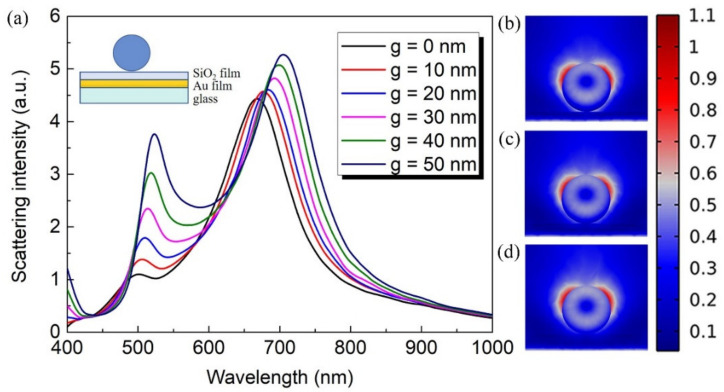
(**a**) The evolution of the scattering spectra for LiNbO_3_ particle of diameter D = 300 nm placed on SiO_2_ film/Au film/glass substrate with an increasing thickness (g) of SiO_2_ layer, g = 0 nm, 10 nm, 20 nm, 30 nm, 40 nm, and 50 nm. (**b**–**d**) represent the electric field distribution in the yz plane for g = 10 nm at 670 nm, g = 30 nm at 690 nm, and g = 50 nm at 705 nm, respectively.

In Figure 5a, we calculate the scattering spectra for a LiNbO_3_ nanoparticle of diameter D = 300 nm located on a SiO_2_ film/Au film/glass substrate with an increasing thickness (g) of the SiO_2_ film. Note that, with the increasing of g, the scattering spectra has a redshift and becomes stronger. The electric field distribution in the yz plane for g = 10 nm at 670 nm, g = 30 nm at 690 nm, and g = 50 nm at 705 nm are depicted in Figure 5b–d, respectively. It is clear to see that the intensity of the hotspot decreases as the thickness of SiO_2_ increases.

**Figure 6:**
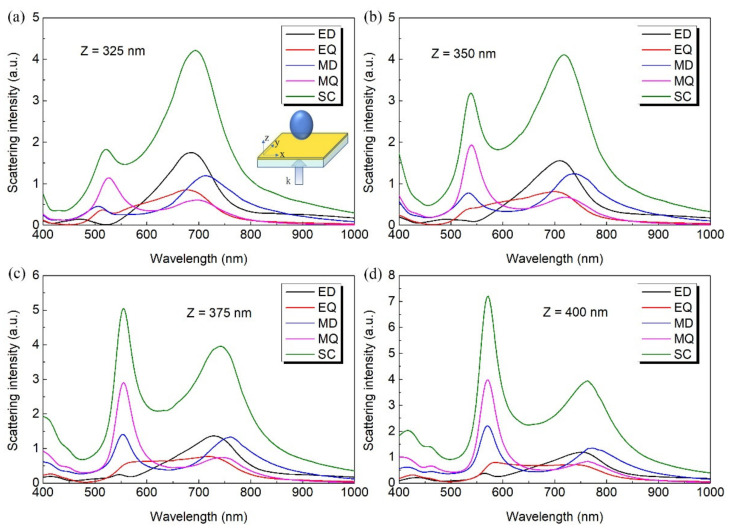
The calculated multipole decomposition of the scattering spectra for LiNbO_3_ particle placed on 50 nm Au film/glass substrate with a fixed axis of 300 nm at in the xy directions and varying axis perpendicular to the Au film. Z represents the length of the particle in the z direction with (**a**) Z = 325 nm, (**b**) Z = 350 nm, (**c**) Z = 375 nm and (**d**) Z = 400 nm.

Furthermore, we studied the influence of the shape of the LiNbO_3_ nanoparticle on the scattering spectra by considering the LiNbO_3_ nanoparticle with its long axis (Z) perpendicular to the Au film. As shown in Figure 6, the two main peaks of the scattering spectra exhibit redshift with the increase of Z. In particular, for all the values of Z in consideration, the coupled dipole resonance slowly damps out as the quadrupole resonance is strongly enhanced.

## 4. Conclusions

We investigated the influence of substrate on the scattering spectra of a LiNbO_3_ nanoparticle by using the FDTD technique. With the increase of the refractive index of the dielectric substrate, the ED resonance is enhanced and MD resonance is weakened. In contrast, the coupling of a LiNbO_3_ nanoparticle to an underlying Au film results in a sharper resonance. It is demonstrated that SiO_2_ film between the LiNbO_3_ particle and the Au film has a significant influence on the scattering spectra. Moreover, the shape of the LiNbO_3_ nanoparticle also affects the scattering spectra. In general, this work demonstrates that the different scattering properties can be obtained by adjusting the structure of the substrate and the nanoparticle. The results presented here broaden the study of moderate-refractive-index dielectric-metal hybrid systems, which may advance the application of LiNbO_3_ nanoparticles in lab-on-chip photonic devices and sensitive biosensors.

## Data Availability

The data presented in this study are available on request from the corresponding author.
